# Supplementary material for: A Review of Probe-Based Enrichment Methods to Inform Plant Virus Diagnostics
Source: Int J Mol Sci. 2024 Jul 30;25(15):8348. doi: 10.3390/ijms25158348 (PMC11312432; doi:10.3390/ijms25158348)
Supplement: Supplementary file 1 [file ijms-25-08348-s001.zip › ijms-3070098-supplementary.pdf]

Supplementary Table S1: Raw data and virus list for Figure 1

O'Flaherty (conserved)*		O'Flaherty (specific)**		Wylie		Briese	
229E	1.38	229E	98.67	Influenza A virus (H3N2)	92.4	Influenza A virus H3N2	3.51
NL63	19.52	NL63	96.11	Influenza B virus	30.35	Middle East respiratory	0.01
TGEV	93.6	OC43	91.44	Human parvovirus	10.2	Enterovirus D68	28.03
OC43	37.05	HKU1	7.4	Norovirus GII-4	51.87	Dengue virus 3	36.43
HKU1	1.36	AdV B11	24.55	Parechovirus 1	7.97	West Nile virus	0.01
MERS-CoV	82.21	AdV C2	1.32	BK polyomavirus	87.06	Ebola virus	0.02
IBV	91.51	AdV E4	90.66	JC polyomavirus	89.89	Cache Valley virus	0.06
AdV A18	79.91	HBoV1	0.25	Human rhinovirus 15	74.06	Herpes simplex virus 1	26.10
AdV B11	48.65	HBoV3	4.71	Human respiratory syncytial virus, type B	86.41		
AdV C2	62.45	HPeV1	45.42	Human herpesvirus 1	0.78		
AdV E4	96.74	HPeV6	99.06	Torque teno virus	58.96		
Parvo B19	97.9	HRV B14	98.99	Human herpesvirus 3	8.33		
HBoV1	2.76	HPIV1	93.46	Torque teno virus	74.38		
HBoV3	7.04	HPIV3	89.73	Human adenovirus B,	10.42		
Reo T1	0.03	HPIV2	98.54	TTV-like mini virus isolate	37.5		
Reo T2	57.11	RSV A2	96.4	Human adenovirus C, type 1	84.68		
Reo T3	34.39	RSV B1	89.83	Torque teno mini virus	22.66		
Rota A	75.23	HMPV75	41.59	Human parainfluenza virus 3	60.35		
Rota B	93.95	HMPV83	93.14	TTV-like mini virus isolate	10.71		
Rota C	56.91	Flu A California/07/2009	81.62	Human bocavirus 1	4.6		
BAV	98.69	Flu A New Caledonia/20/99	92.42	Torque teno virus	14.89		
CTFV	41.56	Flu A Sydney/05/97	99.39	Human adenovirus B, type 3A	75.3		
EHDV	0	Flu Turkey/England/69	93.37	KI polyomavirus	16.63		
GIV	-0.03	Flu A Duck/HongKong	95.81	Human rhinovirus 80	10.94		
BCV	8.05	Flu B Yamagata/16/88	90.07	Human adenovirus C, type 1	99.1		

HPeV1	78.97	Flu B Victoria/87	5.85	Sapovirus	76.69
HPeV6	99.12			Human astrovirus 1	0.06
HRV A2	52.93			Human polyomavirus	70.42
HRV B14	92.94			Torque teno virus	38.17
CPIV2	48.29			Human herpesvirus 6B	37.3
SV41	14.09				
HPIV1	66.23				
HPIV3	57.36				
CDV	73.38				
MV	77.57				
NDV	98.5				
RSV A2	63.02				
RSV B1	71.22				
HMPV75	60.94				
HMPV83	79.69				
Flu A California/07/2009	89.57				
Flu A New Caledonia/20/99	94.55				
Flu A Turkey/Kansas/4880/80	53.31				
Flu A Sydney/05/97	93.84				
Flu A Turkey/England/69	96.33				
Flu A Duck/Hong Kong	99.23				
Flu A Chicken/Hong Kong	95.85				
Flu B Yamagata/16/88	92.7				
Flu B Hong Kong/330/01	24.96				
Flu B Shizuoka/15/01	56.19				
Flu B Victoria/87	47.99				
BKV	0				

This table contains data collected from the raw data in the aforementioned studies in attempt to show the distribution of the range of enrichment [32,45,46]. Additional information for those samples can be found in the original papers.
*O’Flaherty reference samples for conserved panel: a TGEV, transmissible gastroenteritis coronavirus; MERS-CoV, Middle East respiratory syndrome

coronavirus; IBV, avian infectious bronchitis virus; AdV, adenovirus; Parvo B19, human parvovirus B19; HBoV1,3, human bocavirus 1,3; Reo T1,T2,T3, mammalian orthoreovirus type 1,2,3; Rota A, B, C, rotavirus A,B,C; BAV, Banna virus; CTFV, Colorado tick fever virus; EHDV, epizootic hemorrhagic disease virus; GIV, Great Island virus; BCV, Bunyip Creek virus; HPeV1,6, human parechovirus 1,6; HRV, human rhinovirus, CPIV2, canine parainfluenza virus 2; SV41, simian virus 41; HPIV1,3, human parainfluenza virus 1, 3; CDV, canine distemper virus; MV, measles virus; NDV, Newcastle disease virus; RSV, respiratory syncytial virus; HMPV75, human metapneumovirus 75; HMPV83, human metapneumovirus 83; Flu A, influenza A virus; Flu B, influenza B virus, BKV, BK polyomavirus.

**O'Flaherty reference sample for virus specific panel: a AdV, adenovirus; HBoV1,3, human bocavirus1,3; HPeV1,6, human parechovirus1,6; HRV, human rhinovirus; HPIV1,2,3, human parainfluenza virus 1,2,3; RSV, respiratory syncytial virus; HMPV75, human metapneumovirus 75; HMPV83, human metapneumovirus 83; Flu A, influenza A virus; Flu B, influenza B virus [46].
